# Supplementary material for: Seawater-resistant emulsified epoxy resin for effective sand control in unconsolidated sandstone oil reservoir
Source: PLoS One. 2025 Oct 31;20(10):e0335819. doi: 10.1371/journal.pone.0335819 (PMC12578148; doi:10.1371/journal.pone.0335819)
Supplement: Attachment — Submitted filename: Response to Reviewers.docx [file pone.0335819.s004.docx]

**Reviewer #2: Comment 1: References are not correctly mapped in the manuscript in introduction part of the manuscript. Basically, the references are not mapped anywhere in the manuscript.**

*We have revised the references in the Introduction and added references in other parts of the literature. We have optimized the abstract and introduction sections of the paper and added the innovative points of this study compared with similar research.*

In this paper, we study to reduce the negative effect of salt amount on the system by screening a nonionic emulsifying curing agent. Common cationic and anionic emulsifying curing agents do not have this one effect.

**Comment 2: Put the Y-axis title of the FTIR graphs in Figure 9. (a)-(c)**

*We add the definition of the y-axis to the y-axis of the Fourier transform infrared map.(Figure 9 a~c)*

**Comment 3: The novelty of this work needs to be explained in a much better way, It is mentioned in the introduction section that epoxy resin emulsion has been already used for preparing sand consolidating agents. Also the author used the emulsified epoxy resin. Please elaborate the differences in the current composition used in work and previous works by preparing a table showing detailed differences in composition. Additionally, also explain the driving force behind use of similar composition, if one type is already existing in the literatures.**

*We added an overview of the characteristics of chemical sand fixers, comparing the advantages and disadvantages of different chemical sand fixers. In this paper, we study to reduce the negative effect of salt amount on the system by screening a nonionic emulsifying curing agent. Common cationic and anionic emulsifying curing agents do not have this one effect.*

(One of the chemical agent sand consolidation is to use organic or inorganic chemical agent to squeeze into the loose oil layer or pre-filled sand layer, so that the sand particles and sand particles are cemented into a sand layer with a certain degree of strength and good permeability of the sand consolidation process. Currently, chemical agents for sand consolidation include film-forming sand control agent, oil-based resin sand control agent, water-based resin sand control agent, nano-fluid sand control agent, and polymer sand control agent. All these methods have their own advantages and disadvantages, which are summarized in Table 1.)

**Table 1.** Summary of chemical sand fixer characteristics

| Sand Fixing System | Advantages | Disadvantages |
| --- | --- | --- |
| Film-forming sand control agents | Little damage to the stratum, high permeability, excellent in-situ fixation of different particles such as clay, fine sand and filled sand. | High cost and technological monopoly |
| Oil-based resin sand control agent | Wide range of adaptability to temperature gradient, excellent resistance to chemical corrosion, humidity and heat, low price, and strong ability of sand prevention and fixing. | High damage to the formation, difficult to degrade after accidental plugging. |
| Water-based resin sand control agent | It can realize the integration of injection, easy to inject with low viscosity, easy to clean, little damage to the stratum, high strength of the solidification body, and low cost. | The strength of solid sand and the retention rate of permeability can not be achieved at the same time. |
| Nano-fluid sand control agents | Small damage to the stratum, high permeability, can be aggregated again after the consolidated sand body is destroyed, large range of particle size of sand grains captured. | High cost and specific nanoparticle-carrying liquid is required. |
| Polymer sand control agents | Wide reach, high strength of cemented sand (physical and chemical joint action), excellent temperature and salt resistance. | Complex formulation and high cost |

**Comment 4: Provide the detailed composition of the Epoxy Resin (E-51), which would be helpful for the readers for further understanding of the structure and compositions of the resins.**

*We add the main components of the epoxy resin E-51 in the text and describe the basic properties.*

(The main component of epoxy resin E51 is glycidyl ether, with a viscosity of 11,000-14,000 mPa-s at 25°C at room temperature, a molecular weight of 370-380, and an epoxy value of 0.49-0.53.)

**Comment 5: Are the figures 12 and 13 constructed by authors, or taken from literatures. If taken from somewhere, please cite them properly.**

*Figures 12 and 13 are drawn by ourselves.*

**Comment 6: Is the mechanism specified in section 3.2.4 “Sand Consolidation mechanism of emulsion” has been mentioned anywhere in literature, if yes please cite them. If not, how do we agree to this mechanism concretely. Additionally, the slide test for sand and emulsion consolidation method can be a primary test for checking the strength of the system. How does this system actually compares with that of reservoir conditions. How does pressure affect the results of this test.**

*The emulsion sand fixation mechanism described in Section 3.2.4 is described elsewhere in the literature and we have added the references. Aging at 80°C for three days and adding a peripheral pressure of 3 MPa were used to more closely simulate the stratigraphic environment. The final compressive strength will be slightly reduced if no perimeter pressure is added.*

**Comment 7: Further explanation is required for microscopy test in section (Line No 321-335) for better understanding of readers.**

*We explain the results of the microscopic examination in more detail in lines 334-340.*

(As can be seen in Figure 16, the micro-morphology of the cross-section of the sand column was analyzed by metallurgical microscope, and it was found that the resin emulsion-consolidated core possessed abundant pore channels with excellent connectivity between pores. Then the SEM images of the sand column were analyzed, as shown in Fig. 17, the surface of the solidified sand grains was obviously adsorbed with the cured sand fixing agent, which provided support for the excellent mechanical properties of the sand column. Meanwhile, the pore development is excellent, which provides for the permeability retention rate.)

**Comment 8: Conclusion can be enhanced with more focused and conclusive information.**

*We re-summarized the article's conclusions.*

In this study, a new type of epoxy resin emulsion sand curing agent was formulated using epoxy resin E-51 and nonionic emulsion curing agent as the dispersing phase and coupling agent, controlling agent and high salinity water. In order to be able to be used in the field, the curing time of the emulsion-consolidated system and the compressive strength and permeability retention of the consolidated sand columns were evaluated using the indoor experimental evaluation method, and the results are as follows.

(1) When the salt content of the diluent reaches 3.8×10^4^mg/L, the optimal formulation of the system is 16% resin, 24% emulsifying curing agent, 4.8% control agent, and 1% coupling agent. The compressive strength of the solidified core was greater than 3 MPa, the curing time was greater than 12 h, and the permeability retention rate was greater than 75% under the condition of 80 ℃.
(2) By observing the cross section of the sand column through a microscope, the presence of pores can be clearly seen. In addition, through the SEM analysis results, we can also determine that the internal pores of the solidified body are abundant and the connectivity between the pores is relatively good. The sand grains are closely connected to each other through resin.
(3) Through the Fourier infrared diagram, it can be clearly found that the resin reacts with the emulsifying curing agent, which is a good indication that the resin emulsion system has the ability to consolidate the sand particles. Through thermogravimetric analysis, it was found that the mass loss of the consolidated body was extremely low at 250℃.

**Reviewer #1: The aim of this present paper is to develop a new emulsified epoxy resin for sand consolidation by emulsion curing method. While the authors have conducted a series of works, the manuscript lacks deep key discussions, particularly in relation to the paper's key research goals. More importantly, this is not a qualified scientific manuscript with lots of low-level mistakes. For example, the first sentence in Introduction is “Sand control technology is an important measure to ensure stable oil productionError! Reference source not found..”, which is unbelievable in a research paper. I strongly suggest to reject this manuscript.**

*According to Reviewer 1's comments, I have thoroughly revised the paper, corrected several minor errors, and resubmitted it after careful polishing.*

Sand production in oilfield wells is a common issue encountered during oilfield development. It directly or indirectly affects the normal production of the wells. Therefore, it is urgent to adopt appropriate sand control technologies to address the current challenges and ensure high and stable oilfield production.
