# Supplementary material for: Seawater-resistant emulsified epoxy resin for effective sand control in unconsolidated sandstone oil reservoir
Source: PLoS One. 2025 Oct 31;20(10):e0335819. doi: 10.1371/journal.pone.0335819 (PMC12578148; doi:10.1371/journal.pone.0335819)
Supplement: Attachment — Submitted filename: 250816 Review Report.docx [file pone.0335819.s003.docx]

**Article Review**

**Preparation and Evaluation of A New Sand Consolidation Agent: Emulsified Epoxy Resin**

In this article titled Preparation and Evaluation of A New Sand Consolidation Agent: Emulsified Epoxy Resin,” the authors presented a study for development of new type of emulsified epoxy resin with a nonionic emulsified curing agent, fatty amine poly(epoxy ethyl ether), which is able to minimize the effect of salinity. The structure of the emulsified resin and the stability of the curing body were tested, and the effects of curing temperature, time, quartz sand mesh and control agent dosage on compressive strength and permeability were studied. This study seems to be a useful contribution to the literature, and it can be accepted after addressing the following minor issues:

1. The abstract should be improved to enhance flow and to provide the literature gap and the importance of this study. It should be more self-explanatory, especially about the major types of architecture and their relative strengths and weaknesses. It should introduce the field at least in one line and mention the research gap to establish the novelty of this study.
2. The Keywords should be revised to include more relevant keywords and should not include words already mentioned in the title to improve the searchability of the article. For better Search Engine Optimization and searchability, more focused keywords should be included while omitting the generic ones.
3. The English language in this manuscript should be improved. It should also be more elaborate.
4. The methodology section need more explanation.
5. The article should include more relevant literature; it currently consists of very few references to successfully establish the need for the current study.
6. The motivation behind this study should be included with more detail, it should be in proper flow, introducing the gap and research question.
7. The suppositions and boundary conditions should be explicitly declared.
8. The overall flow of language is incoherent, and there are various grammatical and spelling mistakes. These issues should be resolved.

**The End**
